# Manual repositioning of the small bowel and omentum prevents early postoperative small bowel obstruction after laparoscopic colorectal surgery: a propensity score-matched study

**DOI:** 10.1007/s00464-025-12179-1

**Published:** 2025-10-13

**Authors:** Mitsunobu Takeda, Mamoru Uemura, Tsunekazu Mizushima, Shoma Yoshida, Takuya Inoue, Yuki Sekido, Tsuyoshi Hata, Atsushi Hamabe, Takayuki Ogino, Norikatsu Miyoshi, Ichiro Takemasa, Yuichiro Doki, Hidetoshi Eguchi

**Affiliations:** 1 Department of Gastroenterological Surgery, Graduate School of Medicine, The University of Osaka, 2-2 Yamada-Oka, Suita, Osaka 565-0871 Japan; 2 Department of Gastroenterological Surgery, Osaka International Medical & Science Center, Osaka Keisatsu Hospital, Osaka, Japan; 3 Department of Colorectal Surgery, Dokkyo Medical University, Tochigi, Japan

**Keywords:** Early postoperative small bowel obstruction (EPSBO), Anti-adhesion technique, Colorectal surgery, Propensity score matching

## Abstract

**Background:**

Early postoperative small bowel obstruction (EPSBO) is a common complication after colorectal cancer surgery, even with minimally invasive techniques. While anti-adhesion agents are used, effective intraoperative strategies remain limited. We evaluated whether manually repositioning the small bowel and omentum to their anatomical positions (Technique R) at the end of surgery reduces the risk of EPSBO.

**Methods:**

We conducted a two-center, retrospective, propensity score-matched cohort study including 1351 patients who underwent laparoscopic or robot-assisted colorectal cancer surgery between January 2015 and March 2024. Patients were divided into Group A (prior to Technique R, *n* = 770) and Group B (after adoption of Technique R, *n* = 581). One-to-one propensity score matching yielded 488 matched pairs. The primary outcome was the incidence of EPSBO within 30 postoperative days. Multivariate logistic regression was performed to identify independent predictors of EPSBO.

**Results:**

EPSBO occurred in 4.5% of patients in Group A and 1.4% in Group B (*p* = 0.003). In multivariate analysis, the omission of Technique R was identified as an independent risk factor (odds ratio [OR]: 3.09; 95% confidence interval [CI] 1.23–7.67; *p* = 0.016), along with stoma creation (OR: 4.79; *p* = 0.002) and intra-abdominal abscess (OR: 2.84; *p* = 0.029). Technique R was not associated with increased operative time or intraoperative complications.

**Conclusions:**

Omission of Technique R was independently associated with a threefold increase in EPSBO risk. Technique R is a simple, safe, and effective maneuver that significantly improves postoperative outcomes after minimally invasive colorectal surgery and should be considered as a standard operative step.

**Graphical abstract:**

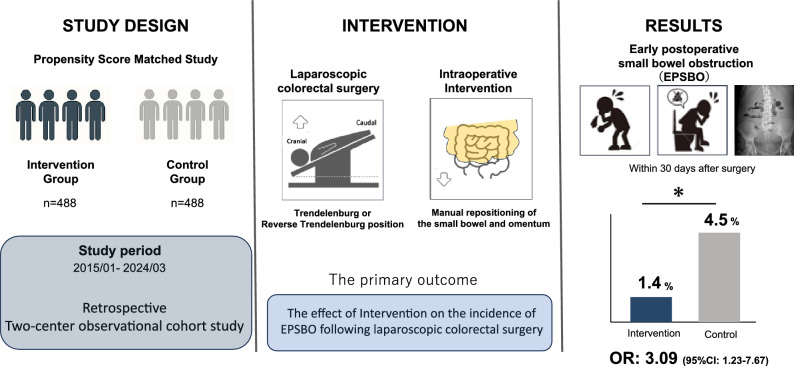

Early postoperative small bowel obstruction (EPSBO) is a frequent and clinically significant complication following colorectal cancer surgery [1]. Despite the widespread adoption of minimally invasive techniques such as laparoscopic and robotic-assisted surgery, as well as routine use of anti-adhesive agents, EPSBO continues to be a major postoperative concern [2, 3]. It is defined as a mechanical or functional obstruction of the small intestine occurring within 30 days after surgery, diagnosed based on clinical symptoms—such as abdominal distension, nausea, vomiting, and prolonged absence of bowel movement—and radiological findings [1, 4–7]. The reported incidence of EPSBO ranges from 4.5% to 15% [2–4, 6, 8–10], and several risk factors have been identified, including advanced age, male sex, low body mass index, prior abdominal surgery, conversion to open surgery, and intra-abdominal infection [2, 4–7, 9, 11–15].

During laparoscopic colorectal surgery, it is standard practice to use gravity in the Trendelenburg or reverse Trendelenburg position to displace the small bowel cranially or caudally and secure the surgical field [16]. However, this displacement alters the physiological positioning of the small bowel and omentum, which may contribute to postoperative adhesion formation and subsequent EPSBO. Although many studies have explored risk factors associated with EPSBO [2, 4–7, 9, 11–15], none have investigated whether manual repositioning of the small bowel and omentum to their anatomical location at the end of surgery (referred to as Technique R) could prevent this complication. While some institutions perform this maneuver routinely based on empirical practice, its clinical efficacy remains unverified.

The aim of this study is to evaluate whether Technique R, a simple intraoperative maneuver, reduces the incidence of EPSBO after minimally invasive colorectal cancer surgery.

To our knowledge, this is the first study to systematically investigate the clinical impact of anatomical repositioning of the small bowel and omentum on EPSBO prevention. We report here the findings of this study, which clarify the effect of Technique R on the occurrence of EPSBO.

## Materials and methods

### Study design

This retrospective, two-center observational cohort study was conducted to evaluate the clinical impact of a specific intraoperative maneuver, hereafter referred to as "Technique R," in patients undergoing minimally invasive surgery for primary colorectal cancer. Eligible patients underwent laparoscopic or robot-assisted colectomy or proctectomy, including ileocecal resection (ICR), right hemicolectomy (RHC), transverse colectomy (T), left hemicolectomy (LHC), sigmoidectomy (S), high anterior resection (HAR), low anterior resection (LAR), abdominoperineal resection (APR), Hartmann’s procedure, and intersphincteric resection (ISR). Patients who required total colectomy for colitic cancer or had multiple synchronous colorectal lesions were excluded.

A total of 1421 consecutive patients who underwent surgery for colorectal cancer between January 2015 and March 2024 at the Osaka International Cancer Institute (formerly Osaka Keisatsu Hospital) and Osaka University were retrospectively reviewed. After excluding 70 patients who were initially scheduled to undergo open surgery, 1351 patients were included in the final analysis. The study protocol was approved by the Institutional Review Boards of both institutions (approval numbers: 1374 and 20163–3). Written informed consent for surgical treatment and the use of clinical data was obtained from all patients prior to enrollment.

### Data source and study population

All data were obtained from the hospital’s electronic medical records and cross-validated using operative and pathology reports. Emergency surgeries were not included. Baseline demographic and clinical data collected included age, sex, body mass index (BMI), ASA physical status, comorbidities, prior abdominal surgery, tumor location, use of preoperative chemotherapy, type of surgery, surgical approach (single-port vs. multiport), stoma formation, prognostic nutritional index (PNI) [17], and pathological TNM stage (UICC 8th edition). There were no missing values in the collected variables.

### Perioperative management and surgical procedures

All patients were diagnosed preoperatively by colonoscopy with biopsy. Tumor location was defined as the distance from the tumor’s distal edge to the anal verge, based on digital rectal and endoscopic examination. Clinical staging was performed using chest and abdominal computed tomography (CT). Neoadjuvant chemotherapy was selectively offered to patients with clinically staged T3 or T4 tumors or node-positive (N1/N2) non-metastatic disease.

Standard oncologic resections were performed according to the principles of total mesorectal excision (TME) for rectal cancer and complete mesocolic excision (CME) for colon cancer [3, 18], in accordance with the guidelines of the Japanese Society for Cancer of the Colon and Rectum. A diverting stoma was created at the surgeon’s discretion in cases of LAR or ISR. For APR and Hartmann’s procedures, permanent stomas were constructed via the intraperitoneal or retroperitoneal route. Operative time was defined from skin incision to skin closure. Conversion to open surgery was defined as any unplanned incision exceeding 7 cm or deviation from the predetermined incision site.

Postoperative complications and mortality were recorded if they occurred during the same hospital admission or within 30 days postoperatively. Complications were categorized using the Clavien–Dindo classification. Adhesion severity was assessed intraoperatively based on the Zühlke classification, which grades adhesions from 0 (none) to 4 (very dense, with high risk of organ injury) [19].

Throughout all procedures, the small intestine and omentum were displaced from the surgical field using gravity in the Trendelenburg or reverse Trendelenburg position. Technique R was defined as the manual repositioning of the small intestine and omentum to their anatomical locations after restoration of the neutral position and completion of all surgical steps. In all cases, after completion of the surgical procedure, the patient’s position was returned from the operative position (typically Trendelenburg with lateral tilt) to the horizontal position. Subsequently, the small intestinal mesentery was manually and carefully realigned to its original anatomical orientation, with particular attention to avoid unnatural twisting or folding. Finally, the greater omentum was gently spread over the small intestine to restore the preoperative intra-abdominal configuration as closely as possible. This procedure was applied uniformly across right hemicolectomy, left hemicolectomy, and low anterior resection. Although the intraoperative positioning differed depending on the type of surgery (Trendelenburg with left tilt for right hemicolectomy, and with right tilt for left hemicolectomy), the final step of returning the patient to the horizontal position and manually restoring the intra-abdominal anatomy remained the same. This maneuver was not employed before 2019 (Group A) but was consistently applied thereafter (Group B). In addition, the mesenteric defect created during right hemicolectomy was not routinely closed; it was left open in all cases. For all right-sided resections, extracorporeal functional end-to-end anastomosis was consistently performed, and the orientation was uniformly isoperistaltic. The use or omission of manual repositioning (Technique R) was clearly documented in all operative reports, and we reviewed the operative notes of all cases to verify its implementation status.

A total of eight colorectal surgeons participated in this study. The adoption of Technique R was implemented in a structured, department-wide manner in 2019, following the appointment of a new chief surgeon. All participating surgeons were certified specialists in gastrointestinal and colorectal surgery and had completed endoscopic surgical certification. Although some staff rotations occurred during the study period, the technical implementation of Technique R was standardized across the department after its formal introduction.

### Outcome measures

The primary outcome was the effect of Technique R on the incidence of early postoperative small bowel obstruction (EPSBO) following minimally invasive colorectal surgery. EPSBO was defined as the presence of clinical symptoms such as abdominal distension, nausea, or vomiting within 30 days postoperatively, along with no passage of stool for over 24 h, and radiological confirmation of dilated bowel loops with multiple air-fluid levels on plain abdominal X-ray or CT scan. While nasogastric tube reinsertion was performed when clinically necessary, it was not used as a diagnostic criterion. In this study, mechanical SBOs and paralytic ileus were not differentiated, as both conditions contribute similarly to early postoperative obstruction in the clinical context. The secondary objective was to identify potential risk factors associated with EPSBO.

### Statistical analysis

Continuous variables were reported as medians with interquartile ranges (IQR), and categorical variables as frequencies and percentages. Pre-matching comparisons were performed using Student’s *t*-test or Wilcoxon rank-sum test for continuous variables and the chi-square test or Fisher’s exact test for categorical variables. A two-tailed *p*-value < 0.05 was considered statistically significant.

To reduce selection bias and adjust for confounding, propensity score matching (PSM) was performed using a 1:1 nearest-neighbor algorithm with a caliper width of 0.2. Propensity scores were derived from a multivariate logistic regression model that included the following 10 covariates considered relevant to surgical complexity: sex, age, BMI, ASA classification, history of abdominal surgery, TNM stage, tumor location (left-sided vs. right-sided), prognostic nutritional index (PNI), precise tumor location, and surgical approach (single-port vs. multiport).

Matched pairs were used to compare the incidence of EPSBO between Group A and Group B. Baseline covariates, including those not used for matching, were assessed using bivariate analysis.

The univariate and multivariate logistic regression analyses were conducted to identify independent predictors of postoperative EPSBO. All statistical analyses were conducted using JMP Pro version 17.0.0 for Mac (SAS Institute Inc., Cary, NC, USA).

Univariate analysis was performed to evaluate the crude association between each variable and the occurrence of EPSBO without adjusting for other covariates. Variables with *p* < 0.05 in univariate analysis were subsequently entered into a multivariate logistic regression model, which allowed adjustment for multiple covariates simultaneously to determine the independent effect of each factor.

## Results

### Baseline patient characteristics

**Fig. 1:**
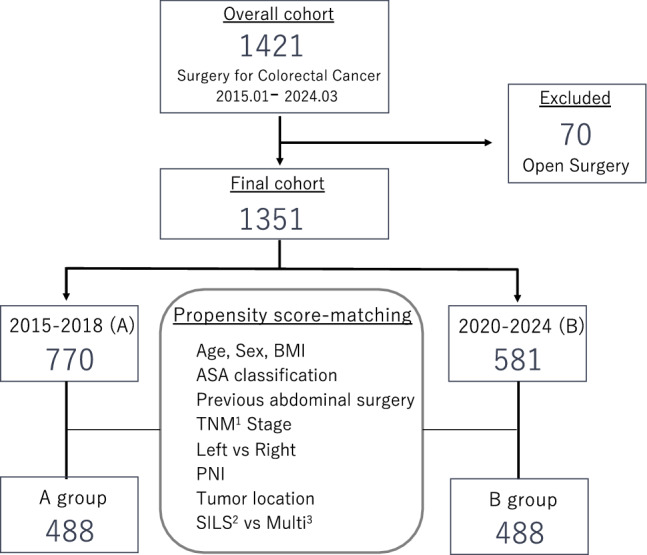
Study design and patient selection flowchart. A total of 1381 patients who underwent open colorectal surgery from 2015 to 2024 were assessed. After exclusions, 1351 patients remained, and propensity score matching yielded 488 patients in each group (2015–2018 vs. 2020–2024)

Between January 2015 and March 2024, a total of 1351 patients underwent laparoscopic or robot-assisted resection for primary colorectal cancer at our institution. Of these, 770 patients treated before the introduction of Technique R were designated as Group A, and 581 patients who underwent surgery using Technique R comprised Group B. After 1:1 propensity score matching, 488 matched pairs (*n* = 976) were included in the final analysis (Fig. 1).

Patient characteristics are summarized in Table 1. Post-matching, baseline characteristics—including age, sex, BMI, ASA classification, tumor location, surgical approach, prior abdominal surgery, prognostic nutritional index (PNI), and pathological TNM stage—were well balanced between the two groups (Table 1). No statistically significant differences were observed for the matched covariates, with all *p*-values > 0.05 and all standardized mean differences < 0.1, indicating adequate covariate balance.

**Table 1:** Patient demographics and clinical characteristics

Characteristics	Overall (*n* = 1351)				Propensity score-matched pairs (*n* = 976)			
	A group *n* = 770	B group *n* = 581	*p*-value	SMD	A group *n* = 488	B group *n* = 488	*p*-value	SMD
Age, median (IQR^a^)	70 (63–77)	72 (64–79)	*0.117*	*0.089*	71 (64–78)	72 (64–79)	*0.618*	*0.045*
Sex, [male/ female], *n*	404/366	326/255	*0.197*	*0.177*	265/223	266/222	*0.877*	*0.037*
BMI^b^, median (IQR)	22.2 (19.8–24.7)	22.1 (19.7–24.7)	*0.759*	*0.017*	22.2 (19.9–24.8)	22.3 (19.7–24.7)	*0.759*	*0.017*
ASA-PS^c^ ≥ 3, *n* (%)	138 (18.0)	86 (14.8)	*0.102*	*0.139*	77 (15.8)	67 (13.7)	*0.696*	*0.077*
TNM^d^ Stage, *n* (%) 0, *n* (%) 1, *n* (%) 2, *n* (%) 3, *n* (%) 4, *n* (%)	23 (2.9) 166 (21.6) 210 (27.3) 268 (34.8) 103 (13.4)	12 (2.0) 130 (22.4) 180 (31.0) 191 (32.9) 68 (11.7)	*0.316*	*0.113*	13 (2.7) 93 (19.1) 143 (29.2) 184 (37.7) 55 (11.3)	10 (2.0) 107 (21.9) 156 (40.0) 154 (31.6) 61 (12.5)	*0.296*	*0.092*
Tumor location, *n* (%) Left side, *n* (%) Right side, *n* (%)	498 (64.7) 272 (35.3)	373 (64.2) 208 (35.8)	*0.861*	*0.112*	309 (63.3) 179 (36.7)	292 (59.8) 196 (40.2)	*0.292*	*0.072*
Surgical approach, *n* (%) SILS^e^, *n* (%) Multi^f^, *n* (%)	444 (57.7) 326 (42.3)	373 (64.2) 208 (35.8)	** < *0.001***	*0.228*	232 (47.5) 256 (52.5)	209 (42.8) 279 (57.2)	*0.226*	*0.098*
Previous abdominal surgery, *n* (%)	255 (33.1)	169 (29.1)	***0.033***	*0.142*	133 (27.3)	122 (25.0)	*0.351*	*0.101*
Diabetes mellitus, [yes/ no], *n*	93 (12.4)	65 (13.3)	*0.656*	*0.118*	57 (11.7)	65 (13.2)	*0.439*	*0.097*
PNI^g^, median (IQR)	48.5 (44.6–52.3)	48.2 (43.6–51.6)	*0.280*	*0.069*	48.2 (44.1–51.9)	47.9 (43.7–51.5)	*0.189*	*0.058*

*P*-values are italicized; bold italics indicate statistical significance (*p* < 0.05)

^a^IQR; Interquartile range, ^b^BMI; Body Mass Index (kg/m^2^), ^c^ASA-PS; American Society of Anesthesiologists physical status, ^d^TNM; Tumor-Node-Metastasis classification, ^e^SILS; Single-incision laparoscopic surgery, ^f^Multi; Multi-port laparoscopic surgery, ^g^PNI; Prognostic Nutritional Index, PNI = 10 × serum albumin (g/dL) + 0.005 × total lymphocyte count (/mm^3^)

### Surgical and perioperative outcomes

As summarized in Table 2, no significant intergroup differences were observed in terms of procedure type (e.g., right hemicolectomy, sigmoid colectomy, low anterior resection), formation of diverting stoma, operative time, blood loss, extent of lymph node dissection, or severity of intra-abdominal adhesions (Zühlke grade ≥ 2) [19].

**Table 2:**
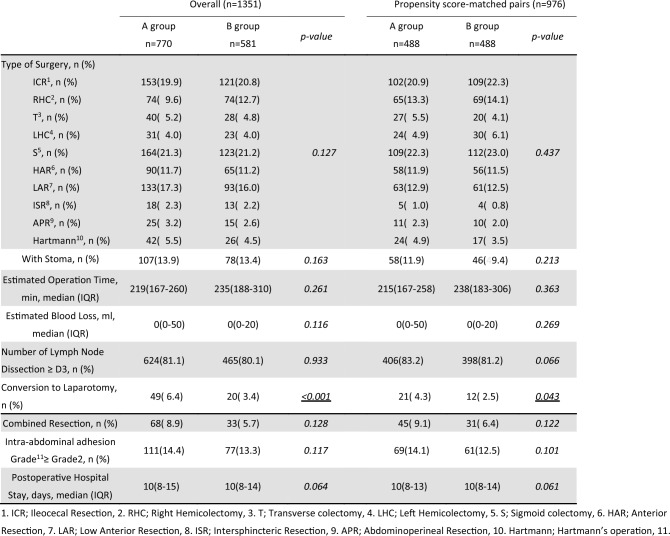
Surgical findings and outcomes before and after propensity score matching

*P*-values are italicized; underlined italics indicate statistical significance (*p* < 0.05)

Even after propensity score matching, the conversion rate to open surgery remained significantly higher in Group A than in Group B (4.3% vs. 2.5%, *p* = 0.043). However, conversion to laparotomy was not identified as an independent risk factor for EPSBO in the subsequent multivariate analysis. The median postoperative hospital stay was 10 days in both groups, without a statistically significant difference (*p* = 0.061).

### Postoperative complications

The overall incidence of postoperative complications was significantly lower in Group B (17.8%) compared to Group A (28.1%). (Table 3). The incidence of intra-abdominal abscess was reduced in Group B (3.3% vs. 7.8%, *p* = 0.023).

**Table 3:**
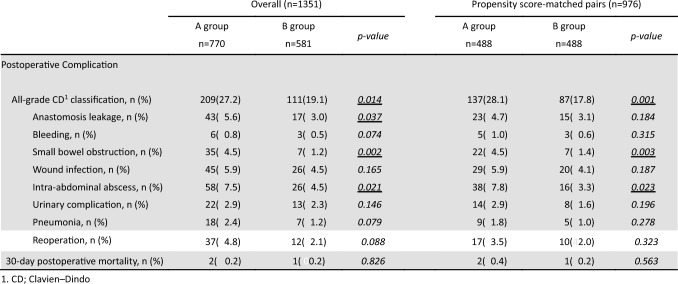
Postoperative complications for before and after propensity score matching

*P*-values are italicized; underlined italics indicate statistical significance (*p* < 0.05)

Especially, Technique R demonstrated a significant protective effect against EPSBO, with a markedly lower incidence in Group B (1.4%) compared to Group A (4.5%, *p* = 0.003).

There were no significant differences between groups in rates of anastomotic leakage, postoperative hemorrhage, surgical site infection, pneumonia, urinary tract complications, or reoperation.

Thirty-day postoperative mortality was low in both groups (0.4% in Group A vs. 0.2% in Group B; *p* = 0.563).

While the reduction in intra-abdominal abscess may have contributed to improved outcomes, the marked decrease in EPSBO suggests a direct effect of Technique R, independent of secondary infectious sequelae. Throughout the study period, our institution did not implement major changes to perioperative management protocols. Enhanced recovery after surgery (ERAS) principles, including early mobilization and early diet advancement, had already been adopted prior to the study period, and surgical site infection (SSI) reduction strategies, such as intraoperative wound irrigation and postoperative antibiotic duration, remained unchanged.

### Risk factors for EPSBO

Table 4 presents the results of univariate and multivariate logistic regression analyses for EPSBO in the matched cohort. Diabetes mellitus was not significantly associated with EPSBO in univariate analysis (*p* = 0.833) and was therefore not retained as an independent predictor in the multivariate model. In multivariate analysis, three independent risk factors for EPSBO were identified: formation of a diverting stoma (odds ratio [OR]: 4.79; 95% confidence interval [CI] 1.76–13.11; *p* = 0.002), intra-abdominal abscess (OR: 2.84; 95% CI 1.12–7.70; *p* = 0.029), and absence of Technique R (Group A) (OR: 3.09; 95% CI 1.23–7.67; *p* = 0.016).

**Table 4:** Univariate and multivariate analyses of risk factors for EPSBO in group A and B patients after propensity score matching

	Univariate analysis			Multivariate analysis		
Variable	OR	95% Cl	*p*-value	OR	95% Cl	*p*-value
Sex [male/female]	2.26	1.02–5.03	***0.044***	1.71	0.73–4.01	*0.216*
Age (year-old), [≥ 70/ < 70]	1.06	0.51–2.24	*0.861*	1.23	0.55–2.76	*0.602*
ASA-PS, [≥ 3/ < 3]	1.52	0.61–3.81	*0.364*			
BMI (kg/m^2)^, [< 20/ ≥ 20]	2.25	0.77–6.54	*0.135*	2.40	0.78–7.35	*0.123*
PNI, [< 45/ ≥ 45]	1.03	0.97–1.07	*0.311*			
Previous abdominal surgery, [ ±]	1.58	0.73–3.39	*0.239*	1.13	0.48–2.68	*0.776*
TNM Stage, [≥ III/ < III	1.07	0.51–2.24	*0.858*			
Tumor location, [Left/Right]	2.44	0.93–6.06	*0.056*	1.45	0.44–4.04	*0.475*
Surgical approach, [SILS/Multi]	0.89	0.37–1.69	*0.559*			
Estimated operation time (min), [per 30 min]	1.24	1.02–1.37	***0.019***	1.91	0.11–9.23	*0.624*
Estimated blood loss (ml), [per 10 ml]	1.01	0.98–1.02	*0.332*			
Conversion to laparotomy, [ ±]	1.10	0.15–6.82	*0.921*			
With stoma, [ ±]	5.61	2.57–12.24	** < *0.001***	4.79	1.76–13.11	***0.002***
Intra-abdominal adhesion grade, [≥ 2/ < 2]	2.79	1.27–6.12	***0.010***	2.18	0.91–5.25	*0.082*
Intra-abdominal abscess, [ ±]	4.88	1.93–12.56	***0.001***	2.84	1.12–7.70	***0.029***
Diabetes mellitus, [yes/ no]	1.07	0.89–2.13	*0.833*			
Treatment, [group A/group B]	3.24	1.38–7.66	***0.007***	3.09	1.23–7.67	***0.016***

*P*-values are italicized; bold italics indicate statistical significance (*p* < 0.05)

Variables with a p-value < 0.20 in univariate analysis were included in the multivariate model. In addition, clinically relevant variables such as age and Previous abdominal surgery were forced into the model regardless of statistical significance

Even after adjustment for these known risk factors, Technique R remained a statistically significant and independent protective factor against EPSBO.

## Discussion

Early postoperative small bowel obstruction (EPSBO) remains a frequent and burdensome complication after colorectal surgery [2–6]. While the adoption of minimally invasive techniques and anti-adhesive materials has improved perioperative outcomes overall [1–4, 8–10], EPSBO continues to delay recovery and prolong hospitalization in a non-negligible proportion of patients [3–5]. In this context, identifying practical, reproducible strategies to mitigate this risk is of significant clinical value.

In our propensity score-matched analysis, we found that simply repositioning the small bowel and omentum to their anatomical position at the conclusion of laparoscopic or robotic colorectal cancer surgery—referred to here as Technique R—was associated with a notably lower incidence of EPSBO. This preventive effect remained significant even after accounting for known risk factors such as the presence of a diverting stoma or intra-abdominal abscess. Importantly, Technique R required no special equipment and minimal additional operative time.

From a mechanistic standpoint, one plausible explanation is that incomplete return of the small bowel to its physiological position after surgery—particularly following prolonged Trendelenburg or reverse Trendelenburg positioning—may promote non-anatomical alignment, predisposing to angulation, kinking, or localized stasis. Indeed, in some cases where reoperation was necessary, we observed the small bowel to have migrated and adhered to the upper abdomen in a configuration that could easily explain obstructive symptoms. Restoring bowel orientation may also enhance postoperative peristalsis and reduce subclinical inflammation contributing to ileus [20].

Its preventive effect persisted regardless of intra-abdominal infection or stoma creation, suggesting that Technique R may directly prevent EPSBO by reducing bowel malposition or minimizing postoperative adhesions and kinking.

Interestingly, we also noted a significantly lower incidence of intra-abdominal abscesses in the group that underwent Technique R. The mechanism by which Technique R may reduce the incidence of intra-abdominal abscess remains speculative. Repositioning the bowel and omentum to their natural anatomical position may help preserve physiological peristalsis and reduce abnormal angulation or folding of the bowel, which could otherwise lead to localized fluid stasis. Additionally, minimizing unnatural contact between the bowel and raw peritoneal surfaces may reduce subclinical inflammation, thereby lowering the risk of abscess formation. Although these explanations are hypothetical, they are consistent with the protective effect observed in our multivariate analysis. Further mechanistic studies are warranted to validate these observations. While this may partially explain the reduction in EPSBO, multivariate analysis suggested that Technique R itself independently contributed to this outcome. This lends further support to the notion that restoring bowel anatomy is not merely an adjunctive step but may actively prevent both mechanical and inflammatory complications. Although diabetes mellitus is known to impair postoperative bowel motility via autonomic neuropathy, it was not a significant risk factor for EPSBO in our cohort. This may reflect the relatively low prevalence of poorly controlled diabetes and the standardized perioperative glycemic management in our institutions.

Our study benefits from a relatively large cohort, consistent surgical practices, and the use of propensity score matching to reduce confounding. However, it is not without limitations. As a retrospective, two-center study, the findings may be influenced by unmeasured biases and local practice patterns. Moreover, EPSBO diagnosis was based on clinical and imaging criteria without direct visualization of adhesions or transition points, and thus the precise mechanism by which Technique R exerts its effect remains speculative. Several potential confounding factors were considered. ERAS, including early feeding and mobilization, had been implemented before the start of the study and remained consistent throughout the study period. Mechanical bowel preparation was routinely performed unless complete obstruction was present. The use of oral laxatives or prokinetic agents was not standardized, but such medications were not systematically administered and were unlikely to differ significantly between groups. All surgeries were performed by certified and experienced colorectal surgeons, and the adoption of Technique R was coordinated at the departmental level, minimizing variability due to surgeon proficiency. Nonetheless, we acknowledge that unmeasured confounding cannot be entirely excluded, and these variables warrant evaluation in future prospective studies.

Despite these caveats, the simplicity and safety of Technique R, combined with its apparent benefit, make it an attractive addition to routine practice. It is an example of how small, thoughtful adjustments during surgery—rooted in anatomical common sense—may yield meaningful improvements in patient outcomes.

## Conclusion

This study suggests that manually repositioning the small bowel and omentum to their physiological location at the end of minimally invasive colorectal cancer surgery (Technique R) is a simple yet effective method for reducing the risk of early postoperative small bowel obstruction. Given its ease of implementation, lack of cost, and potential to improve recovery, we believe Technique R deserves consideration for routine use in laparoscopic and robotic colorectal procedures. Future research should focus on prospective, multicenter studies to validate these findings across diverse surgical settings, as well as mechanistic investigations to elucidate the biological basis of the observed protective effects. Such studies will help determine the generalizability of Technique R and optimize its integration into standardized surgical protocols.

## Declarations

### Research involving human and animal participants

N/A
